# Leech Infestation in the Vulvar Region: Causes of Vaginal Bleeding in a Six years old Child

**DOI:** 10.24248/eahrj.v5i2.664

**Published:** 2021-11-15

**Authors:** Theodomir Sebazungu, Pascaline Kiota Kabungo, Emmanuel Manirakiza, Blaise Dushimiyimana

**Affiliations:** a University of Rwanda, College of Medicine and Health Sciences; b Kabaya District Hospital; c Gisenyi District Hospital; d Ruhengeri referral hospital

## Abstract

Leeches are hermaphroditic rare blood-sucking human endoparasitesof phylum Annelida and class Hirudinea. Leech infestation is a zoonotic disease acquired by drinking contaminated water, swimming in ponds and streams.^1^ Epidemiology of leech bites in literature is limited and the majority of existing data are case reports from the tropics or subtropics.^2–4^

Leech bites can occur on various orifices of the body including internal body cavities and orifices, such as the uterus, rectum, urinary bladder, vulva, nasal cavity, peritoneal cavity, nasopharynx, oropharynx, oesophagus, trachea, bronchi and the vagina.^5^

Different chemicals for leech removal have been utilized and include anesthetics drugs like lidocaine and topical anesthetic spray. Salt, saline, vinegar, alcohol, and heat are also viable options. Of these, saltwater has been shown to be effective in causing the leech to relax and release. Vaginal bleeding resulting from leech bite is rare, but when it occurs, it may be of severe morbidity.^2–4^

In the present case report that happened at Kabaya district hospital, a six year old child with vaginal bleeding that turned out to be caused by vaginal leech infestation is presented.

Kabaya district hospital is a rural hospital with 144-bed capacity and serves 188,902 inhabitants and is geographically difficult to access due to the lack of reliable roads and bridges, especially in the rainy season.

## CASE REPORT

The present case is a six years old female child admitted at Kabaya district hospital, Ngororero district in Northern Province of Rwanda on 19 December 2020 with complaints of pelvic pain and vaginal bleeding for 1 week. The child is 4^th^ child in fiveincluding threegirls and twoboys. She is born from a farming family. Her mother believed that the child was raped by a 10-year-old boy and she was admitted in hospital

On physical examination at admission, she was noted to be with normal anthropometric measures after plotting on the WHO curves weight: 19kg, Height: 107cm. The child was asthenic with the following vital signs: temperature of 36.8°C, heart rate of 112 beats per minute, respiratory rate of 24 cycles per minute, and oxygen saturation of 97% on room air. Capillary refill time was below two seconds, and the extremities were warm. She had conjunctiva and palmo-plantar pallor. There was no lymphadenopathies, no hepatosplenomegaly, no petechiae, no bruises, and no laceration on vulva seen. The rest of the physical exam was unremarkable.

The child was treated with ampicillin IV 300mg three times a day for three days then discharged. Two days after discharge from the hospital, she was readmittedwith profuse vaginal bleeding. Vitamin K5mg IM was prescribed but two days later 26 December 2020, the child was still bleeding and was re-examined during ward round.

**FIGURE 1:**
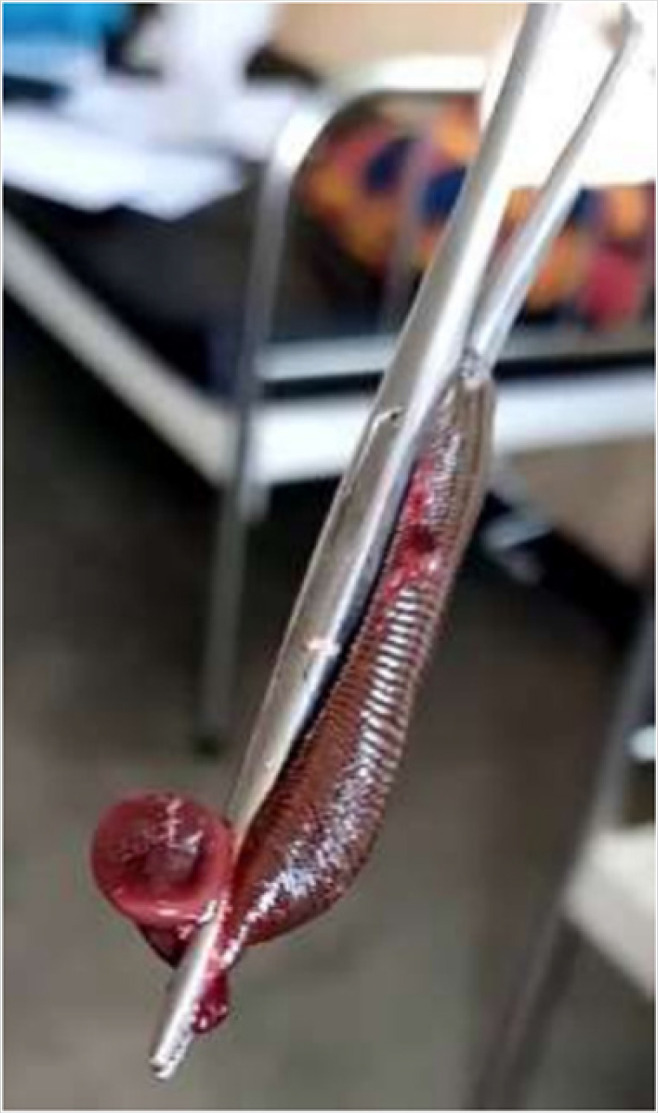
Leech Removed from the Vagina of a Six Years Old Child

Her temperature was 37.4°C, Pulse 104bpm, Saturation: 92% and she had pale conjunctiva. On vagina examination by a general practitioner, a foreign body was seen in the vagina attached on vagina wall and was removed using a forceps. After removal, the foreign body was found to be a live leech of about 5cm in length (Figure 1).

Since then the bleeding subsided. A full blood count done after removing the leech revealed anemia with hemoglobin of 5.9g/dl, hematocrit of 17.8, platelets of 244×10^3^ and the child received three Pediatric units of packed red blood cells. On control full blood count after transfusion, she had hemoglobin of 8.6g/dl and she was discharged on iron supplement. Furthermore on retrospective history taking before discharge from the hospital, the mother reported living near a stream of water that never dries up, that contains parasites similar to the one removed from her child's genitalia and that her child usually plays near the stream.

## DISCUSSION

Globally, leech bites is a rare event and morbidities associated with leeches have been less discussed with most cases reported being from the tropics.^6,7^ **Between January 1, 2004** and **December 31**, 17 cases of leech infestation through body orifices in children were managed. This is a retrospective study on age, sex, route of leech entry, investigation and treatment, and outcome. Results Age ranged from 4.5 to 11 years (mean 6.4 ± 1.8 Locally only one case report of leech bites in upper gastrointestinal tract has been reported so far, and no gynecological case of leech bite has been reported in Rwanda.^8^ Scarcity of data for abnormal vaginal bleeding resulting from leech bite is usually translated into being ignored among the potential differential diagnosis. This was the case in this child since the diagnosis of leech infestation was missed on the first admission, and found when the child was re-admitted in hospital. This case report illustrated the need to consider not only sexual abuse but also possibility of vaginal foreign body in particular leech bite.

## CONCLUSION

As earlier discussed, this case report alertsclinicians to consider leech bite among differential diagnosis while dealing with patients with vaginal bleeding and living near a stream of water containingparasites. Foreign body should be excluded for any pediatric patient suspected to be a victim of rape.
